# The Invasive American Weed *Parthenium hysterophorus* Can Negatively Impact Malaria Control in Africa

**DOI:** 10.1371/journal.pone.0137836

**Published:** 2015-09-14

**Authors:** Vincent O. Nyasembe, Xavier Cheseto, Fatma Kaplan, Woodbridge A. Foster, Peter E. A. Teal, James H. Tumlinson, Christian Borgemeister, Baldwyn Torto

**Affiliations:** 1 International Centre of Insect Physiology and Ecology, Box 30772 Nairobi, Kenya; 2 Center for Medical, Agricultural, and Veterinary Entomology, U.S. Department of Agriculture—Agricultural Research Service, 1700 Southwest 23 Drive, Gainesville, FL 32608, United States of America; 3 Kaplan Schiller Research LLC., PO Box 13853, Gainesville, FL, 32604, United States of America; 4 Department of Evolution, Ecology and Organismal Biology,The Ohio State University,318W 12th Avenue, Columbus, OH 43210, United States of America; 5 Center for Chemical Ecology, Department of Entomology, Pennsylvania State University, 14 University Park, PA 16802, United States of America; 6 Center for Development Research (ZEF), University of Bonn, Walter-Flex-Str. 3, 53113 Bonn, Germany; Instituto de Higiene e Medicina Tropical, PORTUGAL

## Abstract

The direct negative effects of invasive plant species on agriculture and biodiversity are well known, but their indirect effects on human health, and particularly their interactions with disease-transmitting vectors, remains poorly explored. This study sought to investigate the impact of the invasive Neotropical weed *Parthenium hysterophorus* and its toxins on the survival and energy reserves of the malaria vector *Anopheles gambiae*. In this study, we compared the fitness of *An*. *gambiae* fed on three differentially attractive mosquito host plants and their major toxins; the highly aggressive invasive Neotropical weed *Parthenium hysterophorus* (Asteraceae) in East Africa and two other adapted weeds, *Ricinus communis* (Euphorbiaceae) and *Bidens pilosa* (Asteraceae). Our results showed that female *An*. *gambiae* fitness varied with host plants as females survived better and accumulated substantial energy reserves when fed on *P*. *hysterophorus* and *R*. *communis* compared to *B*. *pilosa*. Females tolerated parthenin and 1-phenylhepta-1, 3, 5-triyne, the toxins produced by *P*. *hysterophorus* and *B*. *pilosa*, respectively, but not ricinine produced by *R*. *communis*. Given that invasive plants like *P*. *hysterophorus* can suppress or even replace less competitive species that might be less suitable host-plants for arthropod disease vectors, the spread of invasive plants could lead to higher disease transmission. *Parthenium hysterophorus* represents a possible indirect effect of invasive plants on human health, which underpins the need to include an additional health dimension in risk-analysis modelling for invasive plants.

## Introduction

The changing climatic conditions have negatively impacted on human health through emergence and resurgence of infectious diseases, spread of vector borne diseases to new geographical areas and the spread of invasive plant species [1–3]. The spread of invasive plant species is particularly of interest since they often result in widespread replacement of indigenous flora [4,5]. A particularly notorious example is *Parthenium hysterophorus* (Asteraceae). Native to the subtropics and tropics of North and South America, it has now invaded South, East and Central Africa, Asia and Australia, extensively spreading over both cultivated and pastoral lands [6,7]. The high biotic potential and allelopathic properties of *P*. *hysterophorus* favour its fast spread and replacement of other plant species within new areas of distribution [8–11]. A major concern is the potential toxic effects of *P*. *hysterophorus* on human and livestock health, with some governments such as those in Australia, Uganda and Ethiopia establishing national agencies to help curb its spread [12–14]. The weed grows well in malaria endemic areas of East Africa and was shown to be one of the preferred host plants for the Afrotropical malaria vector *Anopheles gambiae* [15,16]. Although *An*. *gambiae* was shown to be highly attracted to and feed frequently on *P*. *hysterophorus*, there was no evidence of it improving survival and fecundity of these vectors. This raised the question as to what attracts mosquitoes to the plant [15]? In general, little is known about the impact of invasive plants on disease-transmitting arthropods. Knowledge of these interactions can serve as a pre-requisite to better assess the potential contribution of invasive plants to the dynamics of vector-borne diseases and associated public health risks.

The success of mosquitoes as disease vectors is dependent on their prolonged survival, ability to feed on multiple hosts and support pathogen development [17]. Feeding on nectar and honeydew enhances mosquito longevity and also serves as a ready source of energy for flight [18,19]. Evidence of sugar feeding in *An*. *gambiae* continues to accumulate [15,18,20,21], and the significance of sugar availability within *Anopheles* mosquitoes’ localities in relation to their population dynamics and vector potential has gained considerable attention [22–26].

In this study, the contribution of the highly aggressive invasive Neotropical weed *P*. *hysterophorus* and two other adapted plant species that are abundant in malaria endemic regions in western Kenya, *Bidens pilosa* (Asteraceae) and *Ricinus communis* (Euphorbiaceae), to the survival and energy reserves of *An*. gambiae was investigated. Since the chemical analysis of mid-gut contents of mosquitoes fed on the three plant species revealed that they ingested plant specific secondary metabolites, some of which have been shown to be toxic [27–29], we isolated and identified these secondary metabolites from the three plant species as follows: the sesquiterpene lactone parthenin from *P*. *hysterophorus*, the alkaloid ricinine from *R*. *communis*, and the polyyne 1-phenylhepta-1, 3, 5-triyne (henceforth referred to as phenylheptatriyne) from *B*. *pilosa*. The compounds were then tested for their effect on vector survival. The fate of these metabolites once ingested by *An*. *gambiae* was further evaluated by monitoring their presence in the mid-gut at 24, 48 and 72 h post feeding. In addition, four ingested plant sugars detected in the gut of mosquitoes that had fed on the three plant species were identified and quantified to confirm nectar feeding.

## Materials and Methods

### Mosquitoes

Mosquitoes used in this study were obtained from a colony reared at the International Centre of Insect Physiology and Ecology (*icipe*), Duduville campus, Nairobi, established in 2001 from blood-fed and gravid *An*. *gambiae s*.*s*. caught at Mbita Point, western Kenya. Mosquitoes were reared at a mean temperature and relative humidity of 31°C, 52% relative humidity (RH) (day) and 24°C, 72% RH, (night) respectively under reversed diel cycle of light (03:01–15:00) and darkness (15:01–03:00). The adults were maintained on a diet of human blood three times per week, along with glucose (6% solution *ad libitum*) (Sigma^®^) continuously available on filter paper. Feeding the mosquitoes on human subjects was approved by the Kenya National Ethical Review Board (protocol number KEMRI/RES/7/3/1), which is renewed on an annual basis. Informed consent was obtained from volunteers working on the project before participating in blood feeding. Fully engorged females were allowed to lay eggs on funnel-shaped filter papers placed over oviposition cups (4 cm diameter, 2 cm depth) inside the cages. Eggs were collected and dispensed into plastic trays (25 cm long × 20 cm wide × 14 cm high) filled to a depth of 8 cm with distilled water. Upon hatching, larvae were reared in these trays at densities of 100–150/ tray and fed fish food (Tetramin^®^, Melle, Germany) three times daily (the total amount of food provided was 0.3 g Tetramin/100 larvae/day). Pupae were collected from rearing trays and transferred to standard 30 × 30 × 30 cm mesh-covered cages with access to water and 6% glucose solution *ad libitum*. Newly emerged adult females intended for use in bioassays were provided with watered cotton pads (no access to glucose solution or blood meal).

### Plant species

**Fig 1:**
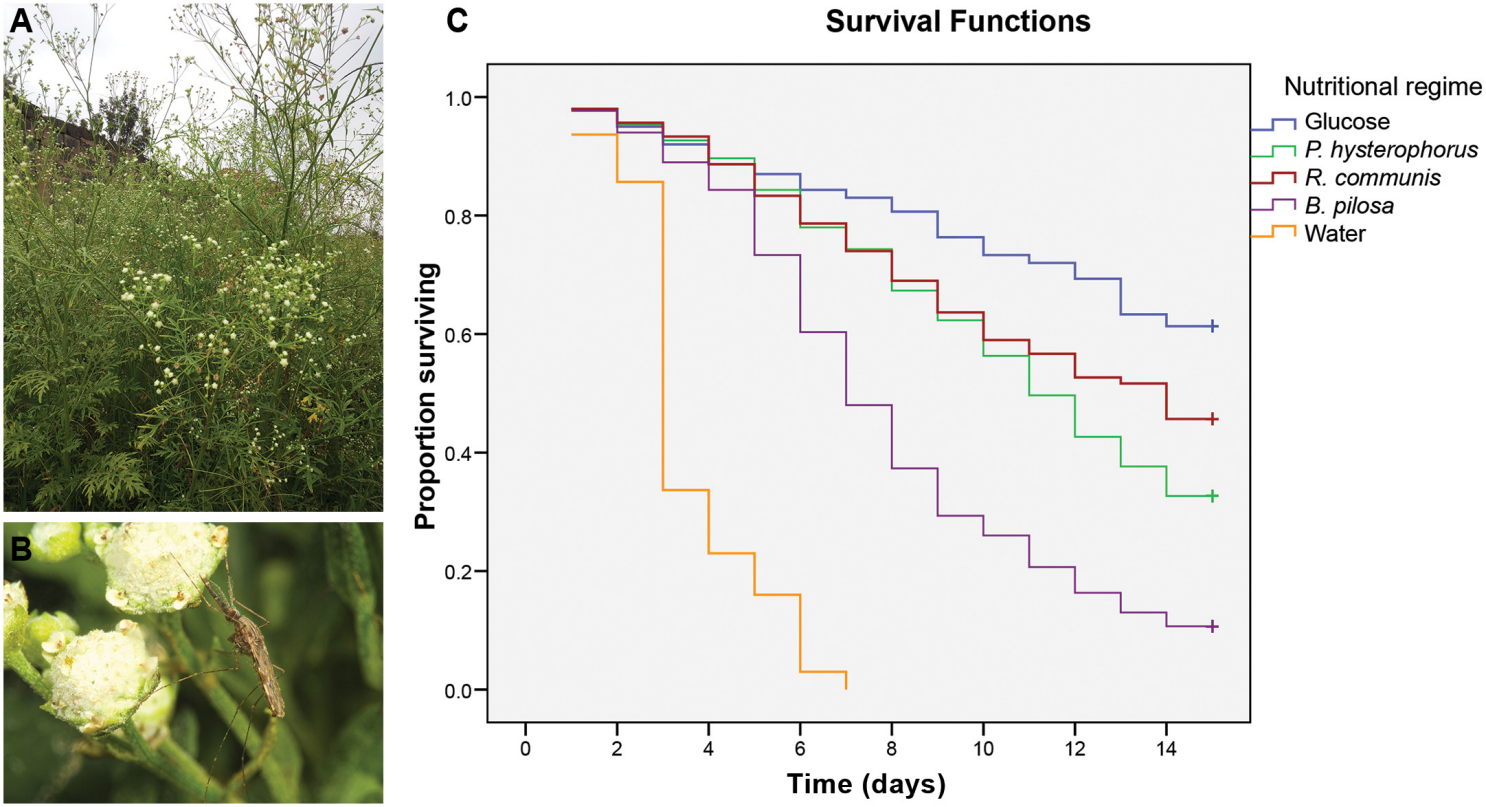
Probing and survival on intact plant. A) Invasive weed, *Parthenium hysterophorus*, B) Female *Anopheles gambiae* probing on the flowers of *P*. *hysterophorus*, and C) Proportion of female *An*. *gambiae* surviving after exposure to different nutritional regimes. Glucose solution (6%) and water were used as positive and negative controls, respectively. The surviving mosquitoes from each treatment were censored on day 15 (survival curves marked + symbol).

The three plant species were selected, based on four criteria: 1) they are abundant in malaria-endemic regions of western Kenya, which had formed the basis of their selection in previous studies by Impoinvil et al. [20] and Manda et al. [15]; 2) based on a study by Manda et al. [26], which showed that *P*. *hysterophorus* (Fig 1A) was highly preferred by *An*. *gambiae* but did not support survival or fecundity, we selected both *P*. *hysterophorus* and *R*. *communis* (which had the opposite effect), to understand their contribution to the mosquito energy reserve; 3) the study by Manda et al. [26] speculated potential fitness-related benefits by *An*. *gambiae* from anti-plasmodial metabolites in *P*. *hysterophorus*, hence we selected *B*. *pilosa*, which belongs to the same family as *P*. *hysterophorus*, and has also been shown to have anti-plasmodial activity [27], for comparison; 4) since the major secondary metabolite in *P*. *hysterophorus* has been shown to have toxic effects on humans and animals [28], we selected *R*. *communis*, which also contains toxic metabolites to humans and animals [29], and *B*. *pilosa*, which has no known toxic effects and is consumed as a vegetable in Kenya [30]; these last two plants provided suitable candidates for comparison of toxic effects of *P*. *hysterophorus* metabolites on *An*. *gambiae*. Besides, our previous study had shown that *R*. *communis* had a high sugar content (6.1–7.6 μg/mg), followed by *P*. *hysterophorus* (2.5–4.7 μg/mg), while *B*. *pilosa* had the lowest sugar content (1.8–2.4 μg/mg) [31]. The three plants were identified with the aid of botanists from the National Museum of Kenya (Voucher numbers 2011/105, 2011/107 and 2011/108 for *B*. *pilosa*, *R*. *communis* and *P*. *hysterophorus*, respectively). Free growing plant seedlings in Duduville-Nairobi campus of *icipe*, were transplanted into potted soil enriched with compost, watered daily, and maintained in a screen house under ambient environmental conditions. Plants were used at the flowering stage.

### Survival assays

Batches of newly emerged (1-day old) mosquitoes, each consisting of 200 females, were placed in 60 × 60 × 60 cm cages. Randomly selected groups of mosquitoes were allowed access to one of the selected potted, caged plant species. Since mosquitoes are known to probe plants for sugars [15, 23–25], we investigated the contribution of sugars to mosquito survival using probing assays (Fig 1B). In the control groups, batches of 200 females were allowed continuous access either to a 6% (wt:vol) glucose solution in filter-paper wicks (positive control) or water pads only (negative control). The experimental setup was maintained under the rearing conditions in terms of temperature and RH (day, 31°C, 52% RH and night, 24°C, 72% RH). Mosquitoes in each cage had access to one of the three plant species and to cotton pads wetted with distilled water. Potted plants, glucose solution, and water pads were changed every two days. The survival of female mosquitoes kept on different nutritional regimes was monitored for 14 days. Dead mosquitoes were removed daily and counted. Mosquitoes were sampled at day 7 (when there were few mosquitoes still surviving on water diet) and analyzed for energy reserves. All tests were replicated five times.

### Extraction and detection of specific mosquito ingested plant metabolites

A sample of 30 mosquitoes was obtained from each of the five replicates for all five food regimes on day 7. Preliminary studies had indicated that parthenin and phenylheptatriene, the major metabolites in *P*. *hysterophorus* and *B*. *pilosa*, respectively, dissolve in dichloromethane, while ricinine, which is dominant in *R*. *communis*, is soluble in acetone. Therefore, mid-guts were dissected from females and extracted in dichloromethane for those held on *P*. *hysterophorus* and *B*. *pilosa* and acetone for those held on *R*. *communis* for 30 min. The mid-gut extracts of mosquitoes held on *P*. *hysterophorus* and *B*. *pilosa* were analysed by coupled gas chromatography-mass spectrometry (GC-MS) while those from the *R*. *communis* group were analysed by coupled liquid chromatography-electrospray ionisation mass spectrometry (LC-ESMS). GC-MS analysis was carried out in the splitless injection mode using an Agilent Technologies 7890 gas chromatograph coupled to a 5975C inert XL EI/CI mass spectrometer (EI, 70 eV, Agilent, Palo Alto, CA) equipped with an HP-5 column (30 m × 0.25 mm ID × 0.25 μm film thickness, Agilent, Palo Alto, CA). Helium was used as the carrier gas at a flow rate of 1.2 ml/min. The oven temperature was held at 35°C for 3 min, then programmed to increase at 10°C/min to 280°C and maintained at this temperature for 10 min. The mid-gut profiles were compared with the corresponding profiles of flower and leaf extracts and the matching peaks identified through comparison of their mass spectra with library data [32].

The LC-ESMS used consisted of a quaternary LC pump (Model 1200) coupled to Agilent MSD 6120-Single quadrupole MS with electrospray source (Palo Alto, CA). The system was controlled using ChemStation software (Hewlett-Packard). Reverse-phase liquid chromatography was performed using an Agilent Technologies 1200 infinite series LC, equipped with a Zorbax Eclipse Plus C_18_ column, 4.6 x 100 mm x 3.5 μm (Phenomenex, Torrance, CA), using an isocratic program 40% A (5% formic acid in LC-grade ultra pure H_2_O): 60% B (LC-grade acetonitrile) (Sigma, St. Louis, MO) at a flow rate of 1ml min^-1^. Injection volume was 10 μl, and data were acquired in a full-scan positive-ion mode using a 100 to 800 *m/z* scan range. The dwell time for each ion was 50 ms. Other parameters of the mass spectrometer were as follows: capillary voltage, 3.0 kV; cone voltage, 70 V; extract voltage, 5 V; RF voltage, 0.5 V; source temperature, 110°C; nitrogen gas temperature for desolvation, 380°C; and nitrogen gas flow for desolvation, 400 L/h.

Parthenin, phenylheptatriyne and ricinine were identified by chromatographic and spectroscopic techniques (see below).

### Extraction and identification of plant toxins from host plants

Leaves and flowers of *P*. *hysterophorus*, *R*. *communis* and *B*. *pilosa* were separately air-dried and crushed using an electric grinder (Retsch GmbH, Haan, Germany). The air-dried material (1 kg each) was extracted with methanol (2.5 L) in a Soxhlet apparatus for 48 h and concentrated *in vacuo* to give 90, 83 and 70 g extracts for *P*. *hysterophorus*, *R*. *communis* and *B*. *pilosa*, respectively. These extracts were chromatographed on silica gel (32–63 μm Riedel-De Haen 31607) using a hexane-ethyl acetate gradient. The parthenin mix from *P*. *hysterophorus* was eluted with 70% hexane: ethyl acetate and further purified by column chromatography as described above using hexane-acetone gradient to give parthenin (1.2g). The ricinine mix from *R*. *communis* was similarly eluted with 60% hexane-ethyl acetate, and upon further chromatography, using a hexane-acetone gradient, gave ricinine (500 mg). The phenylheptatriyne mix from *B*. *pilosa*, eluted with 100% hexane, was further purified by column chromatography and recrystallization from hexane-methanol to give phenylheptatriyne (50 mg).

Two-to-five milligrams of isolated compounds parthenin, ricinine and phenylheptatriyne were each dissolved in CDCl_3_ (Cambridge Isotope Laboratories, Inc., Tewksbury, MA), and placed in 2.5 mm NMR tubes (Norell Inc., Marion, USA). All NMR spectra were acquired at 22°C using a 5 mm TXI cryoprobe (Bruker Corporation, Billerica, MA) and a Bruker Avance II 600 console (600 MHz for ^1^H and 151 MHz for ^13^C), except for 2D NOESY data, which were collected at 25°C. Residual CHCl_3_ was used to reference chemical shifts to δ (CHCl_3_) = 7.26 ppm for ^1^H, and δ C-6 of parthenin was referenced to 78.5 ppm for ^13^C according to Shimoma et al. [33]. NMR spectra were processed using Bruker Topspin 2.0 and MestReNova (Mestrelab Research, Santiago de Compostela, Spain) software packages. Parthenin is numbered according to Herz et al. [34], Shimoma et al. [33] and Das et al. [35]. The GC-MS analysis was carried out on an Agilent instrument using a HP-5 column as specified above, while LC-ESMS analysis was carried out in the positive-ion mode LC-ESMS using ACE AQ C18 octadecyl-bonded phase column.

### Feeding assays with pure isolates

Parthenin, ricinine and phenylheptatriyne, isolated from the respective host plants as described above, were separately tested in survival assays by feeding 200 female *An*. *gambiae* on 0.04 mg/ml (the natural amount of parthenin detected in the flowers of *P*. *hysterophorus*, hence standardized across for comparison purposes) of each compound dissolved in 3 ml acetone and mixed with 17 ml of 6% glucose solution. Two sets of 200 mosquitoes, were separately fed on 6% glucose solution and 3 ml acetone +17 ml of 6% glucose solution positive controls, while a different set, fed on distilled water, served as a negative control. Mosquitoes were allowed to feed *ad libitum* and observed for seven days. Mortality was recorded daily for seven days and the treatments replicated five times.

A second set of assays with similar treatment as above was conducted to evaluate the fate of the three metabolites once ingested by the mosquitoes. In these assays, the secondary metabolite solutions were removed after 24 h and replaced with 6% glucose solution. Fifty mosquitoes from each treatment were sampled after 24, 48 and 72 h post commencement of the feeding assays, extracted in either dichloromethane (parthenin and phenylheptatriene diets), or acetone (ricinine diet) for 30 min and analysed by GC-MS and/or LC-ESMS for the specific secondary metabolites and their derivatives, replicated three times.

### Analysis of energy reserves

To measure the contributions of each food regime to the energy reserves of the mosquitoes, 30 female *An*. *gambiae* fed on the intact plants as well as the controls were analysed for sugar, glycogen and lipid contents using procedures adapted from Van Handel and Day [36]. Female *An*. *gambiae* were transferred, one at a time, to 1.5-ml microcentrifuge tubes and crushed with a glass rod in 0.2 ml of 2% sodium sulphate solution. An aliquot (0.8 ml) of chloroform-methanol (1:1) was added to wash the glass rod. The tubes were then vortexed and centrifuged at 3,000 rpm for 1 min, and the supernatant was transferred to a clean centrifuge tube. The pellet was retained for glycogen analysis. The supernatant was further subjected to separation by adding 0.6 ml deionised water to it, mixed and centrifuged at 3,000 rpm for 1 min. The upper layer was separated for sugar analysis while the bottom portion was used for lipid analysis.

The fractions for sugar and glycogen analysis were heated at 100°C to evaporate the solvent down to 0.1–0.2 ml level. Anthrone reagent was added to 5 ml level, heated for 17 min at 100°C using water bath (IKA-Combitherm HCB, Landwirtschaftskammer, Rheinland, Bonn, Germany) and allowed to cool. Absorbance was read at 625 nm using a spectrophotometer (Shimadzu Corporation, Kyoto, Japan). For quantification, a stock of 100 mg per 100 ml of anhydrous glucose in deionised water and analysed in triplicate at 12.5, 25, 50, 100 and 150 μl of the prepared solution, was used, following the procedures above to generate a calibration curve. This curve was used to quantify the sugar and glycogen levels in mosquitoes fed on different nutritional regimes.

Lipid content was estimated according to Van Handel [37]. For lipid analysis, all tubes were heated at 90°C until the solution completely evaporated. Samples were then dissolved in 0.2 ml of sulfuric acid and heated at 90°C for 10 min. The tubes were cooled on ice and vanillin-phosphoric acid reagent was added to bring the solution to 5 ml; thereafter the sample in each tube was left at 25°C for 30 min, and then the absorbance was read at 625 nm with a spectrophotometer. Corn oil was used as the standard lipid. For quantification, a stock of 100 mg per 100 ml of corn oil in chloroform, and triplicates of 25, 50, 100, and 200 μl of the solution were analysed using the procedures above to generate a calibration curve. This curve was used to quantify the lipid levels in mosquitoes fed on the different nutritional regimes.

To ascertain the type and amount of sugars ingested by mosquitoes when feeding on different food regimes, 30 female mosquitoes from each of the food regimes, i.e., 6% glucose solution, *R*. *communis*, *P*. *hysterophorus*, *B*. *pilosa*, and water, were further analyzed for specific plant sugars identified in our previous work [31]. Newly emerged female *An*. *gambiae* were held on these food regimes for 24 hours then individual mid-guts dissected and macerated slowly in 1 ml pyridine (Sigma®) for 3 days. These were then derivatised with 100 μl pyridine and 100 μl N-Methyl-bis trifluoro acetamide (MBTFA) (Sigma®) at 60°C for 1 h. The products were subjected to GC-MS analysis under the conditions described above. Plant sugars were identified by comparison of spectra of their trimethylsilyl derivatives with library data [32], and with those of authentic standards. The amount of each of the six sugars present in each mosquito was quantified, based on peak-area comparison with those of authentic standards.

### Chemicals

Synthetic standards of sugars used included: D-(+)-glucose (Sigma, 99%), maltose (Sigma, 99%), sucrose (Sigma, 99.5%), and mannose, (Sigma, 99%).

### Statistical analysis

Survival analysis was carried out using Kaplan-Meier and log-rank tests to compare survival curves and test whether the survival rates differed between the different nutritional regimes. Survival indices were derived by comparing the mean survival of mosquitoes on the other food regimes to that of water only. The differences in the energy reserves of mosquitoes fed on the different food regimes were analysed using the Kruskal-Wallis test and means separated with the two-sample Wilcoxon test. The differences in amounts of specific sugars ingested were detected using one-way ANOVA and Tukey’s post hoc test. The statistical analyses were carried out using R statistical software [38].

## Results

### *Parthenium hysterophorus* improves mosquito survival

Pair-wise comparisons revealed that median survival time differed significantly among the five food regimes (*W* = 1958.66, df = 4, *P* < 0.001), with mosquitoes surviving longest on 6% glucose solution (medium time >14 days), followed by *R*. *communis*, *P*. *hysterophorus*, *B*. *pilosa*, and water, in decreasing order (median times 14, 11, 7 and 3 days, respectively) (Fig 1C, Table 1). The survival curves of the five nutritional regimes also differed significantly (Log Rank = 2342.958, df = 1, *P* < 0.001).

**Table 1:** Survival times in days of *An*. *gambiae* exposed to different nutritional regimes.

Nutritional regime	N	Median	Mean	± SE	Survival index
Water	1000	3.00	3.55	0.06	1.00^e^
Glucose solution	1000	-	12.24	0.18	3.45^a^
*P*. *hysterophorus*	1000	11.00	10.61	0.17	2.99^c^
*R*. *communis*	1000	14.00	11.10	0.18	3.12^b^
*B*. *pilosa*	1000	7.00	8.00	0.16	2.25^d^

N = total number of mosquitoes assayed, SE = standard error, - = median not estimated, number followed by different letter superscript in the survival index column differ significantly (*P* < 0.05). The surviving mosquitoes from each treatment were censored on day 15.

**Fig 2:**
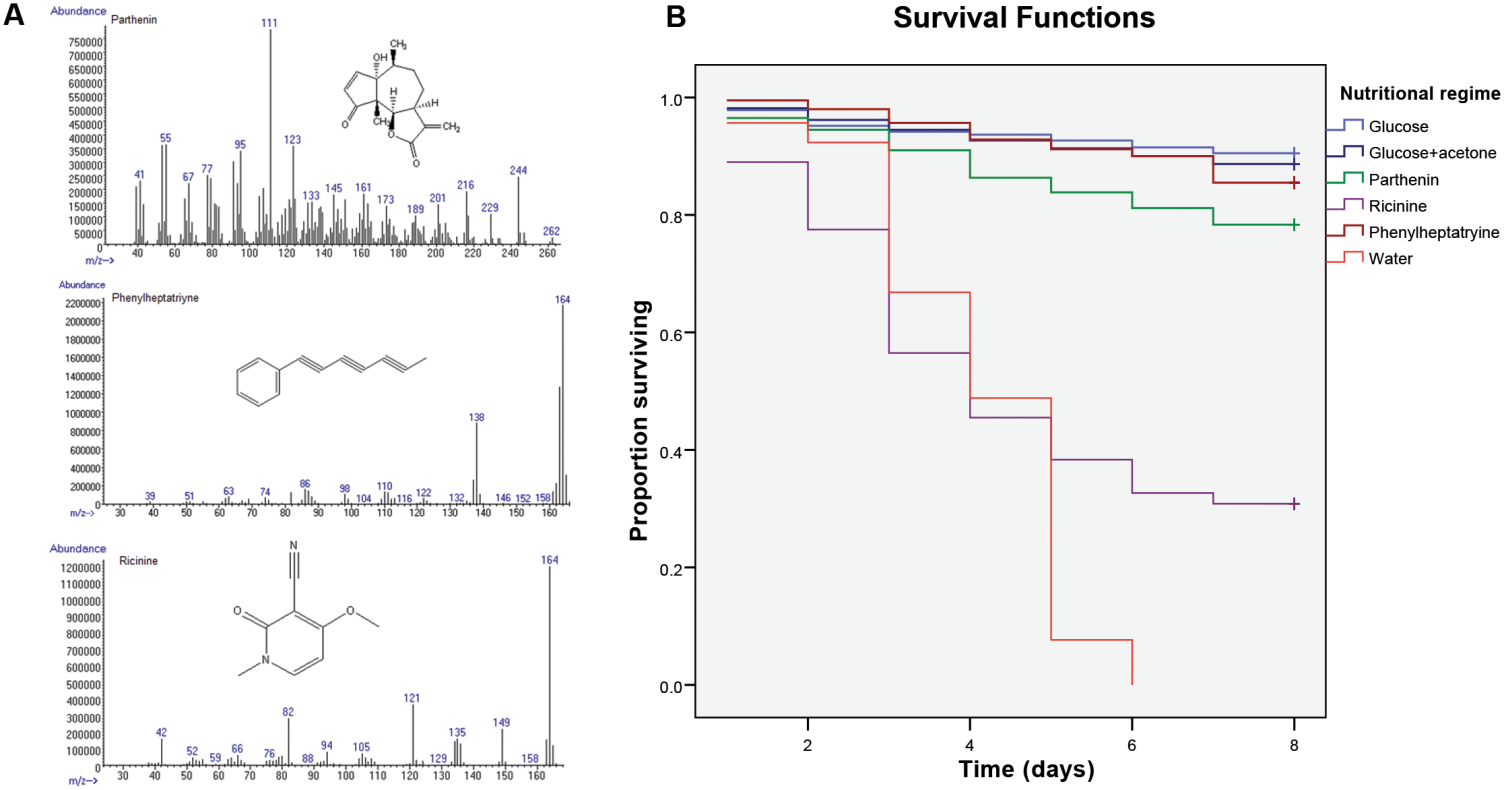
Major secondary metabolites and their effect on *An*. *gambiae* survival. A) Mass spectrum and chemical structures of three known plant metabolites detected in the mid-guts of mosquitoes: parthenin from *P*. *hysterophorus*; ricinine from *R*. *communis*, and phenylheptatriyne from *B*. *pilosa*; B) Proportion of female *An*. *gambiae* surviving after exposure to different plant toxins detected in mosquito mid-guts. Glucose solution (6%) and water were used as positive and negative controls, respectively, with the toxins dissolved in 6% glucose solution. The toxins were presented dissolved in 6% glucose solution and acetone. The surviving mosquitoes from each treatment were censored on day 8 (survival curves marked + symbol).

GC-MS and LC-ESMS analyses of the mid-gut contents of surviving mosquitoes held on the three plant species revealed that they ingested major secondary metabolites from the plants besides the common plant sugars. These included the sesquiterpene lactone parthenin from *P*. *hysterophorus*, the alkaloid ricinine from *R*. *communis*, and polyyne1-phenylhepta-1, 3, 5-triyne from *B*. *pilosa* (Fig 2A). None of these compounds were detected in the mosquitoes fed on the positive and negative controls.

We isolated and further verified the structure of each toxin from leaf and flower extracts by NMR, as follows:

Parthenin: ^1^H NMR (600 MHz, Chloroform-*d*): δ 7.53 (d, *J* = 5.9 Hz, 1H), 6.17 (d, *J* = 5.9 Hz, 1H), 5.00 (d, *J* = 7.9 Hz, 1H), 3.50 (m, 1H), (1.23 (3H, s), 1.11 (d, *J* = 7.2 Hz, 3H). ^13^C NMR (151 MHz, CDCl_3_): δ 210.5 (C-4), 170.7 (C-12), 162.8 (C-2), 140.2 (C-11), 131.7 (C-3), 121.6 (C-13), 84.3 (C-1), 78.5 (C-6), 58.9 (C-5), 44.0 (C-7), 40.5 (C-10), 29.6 (C-9), 28.1 (C-8), 18.1 (C-15), 17.2 (C-14). Proton chemical shifts 1.28 and 1.11 for methyl at C-5 and at C-10 are consistent with [34], respectively. Additionally, carbon and proton chemical shifts are in agreement with previous reports [33,35,39] for the presence of parthenin.

Phenylheptatriyne: ^1^H NMR (600 MHz, Chloroform-*d*) δ 7.53–7.49 (m, 2H), 7.39–7.35 (m, 2H), 7.34–7.30 (m, 1H), 2.01 (s, 3H).^13^C NMR (151 MHz, CDCl_3_) δ 132.96, 129.49, 128.45, 121.14, 78.24, 77.23, 77.02, 76.81, 75.19, 74.62, 67.41, 64.91, 58.96, 29.72. These proton and carbon chemical shifts are consistent with Changa et al. [40].

Ricinine: ^1^H NMR (600 MHz, Chloroform-*d*) δ 7.53 (d, *J* = 7.7 Hz, 1H, H-6), 6.08 (d, *J* = 7.7 Hz, 1H, H-5), 4.00 (s, 3H, OCH_3_), 3.55 (s, 3H, NHCH_3_). ^13^C NMR (151 MHz, CDCl_3_) δ 172.49 (C-2), 161.41 (C-4), 143.59 (C-6), 113.78 (CN), 93.66 (C-5), 88.82 (C-3), 57.25 (OCH_3_), 37.73 (NCH_3_). Proton and carbon chemical shifts are consistent with Ramos-López et al. [41] and Souza et al. [42]. GC/MS data: m/z 164 (molecular ion and base peak), 149, 134, 121, 94, 82 and 66. LC-MS data: m/z 165 (molecular ion and base peak), 187 [M + H^+^ + Na^+^]^+^, 329 [M + H^+^ + M^+^]^+^ and 351 [M + H^+^ + M^+^ + Na^+^].

There was a significant difference in the survival time among the three plant isolates and the controls (*W* = 831.55, df = 5, *P* < 0.001). Survival was lowest on water and ricinine followed by parthenin, phenylheptatryine, and glucose solution, in that order (Fig 2B, Table 2). There was no significant difference detected between glucose and glucose + acetone diets (*P* = 0.315) (Table 2). Log rank analysis also showed a significant difference in the survival curves of the five nutritional regimes (Log Rank = 712.94, df = 1, *P* < 0.001).

**Table 2:** Survival times in days of *An*. *gambiae* exposed to plant toxins.

Nutritional regime	N	Median	Mean	± SE	Survival index
Water	600	4.00	4.11	0.05	1.00^e^
Glucose solution	600	-	7.55	0.06	1.84^a^
Glucose + acetone	600	-	7.52	0.06	1.83^a^
Parthenin	600	-	7.12	0.08	1.73^c^
Ricinine	600	4.00	4.70	0.11	1.14^d^
Phenylheptatriyne	600	-	7.53	0.06	1.83^b^

N = total number of mosquitoes assayed, SE = standard error, - = median not estimated, number followed by different letter superscript in the survival index column differ significantly (*P* < 0.05). The secondary metabolites were presented in 6% glucose solution. The surviving mosquitoes from each treatment were censored on day 8.

Further analysis revealed that the secondary metabolites were detectable in the mid-guts of *An*. *gambiae* 24 h after the commencement of feeding assays but were not present 48 h later. No derivatives of the compounds were detected.

### Food source modulates mosquito energy reserves

**Fig 3:**
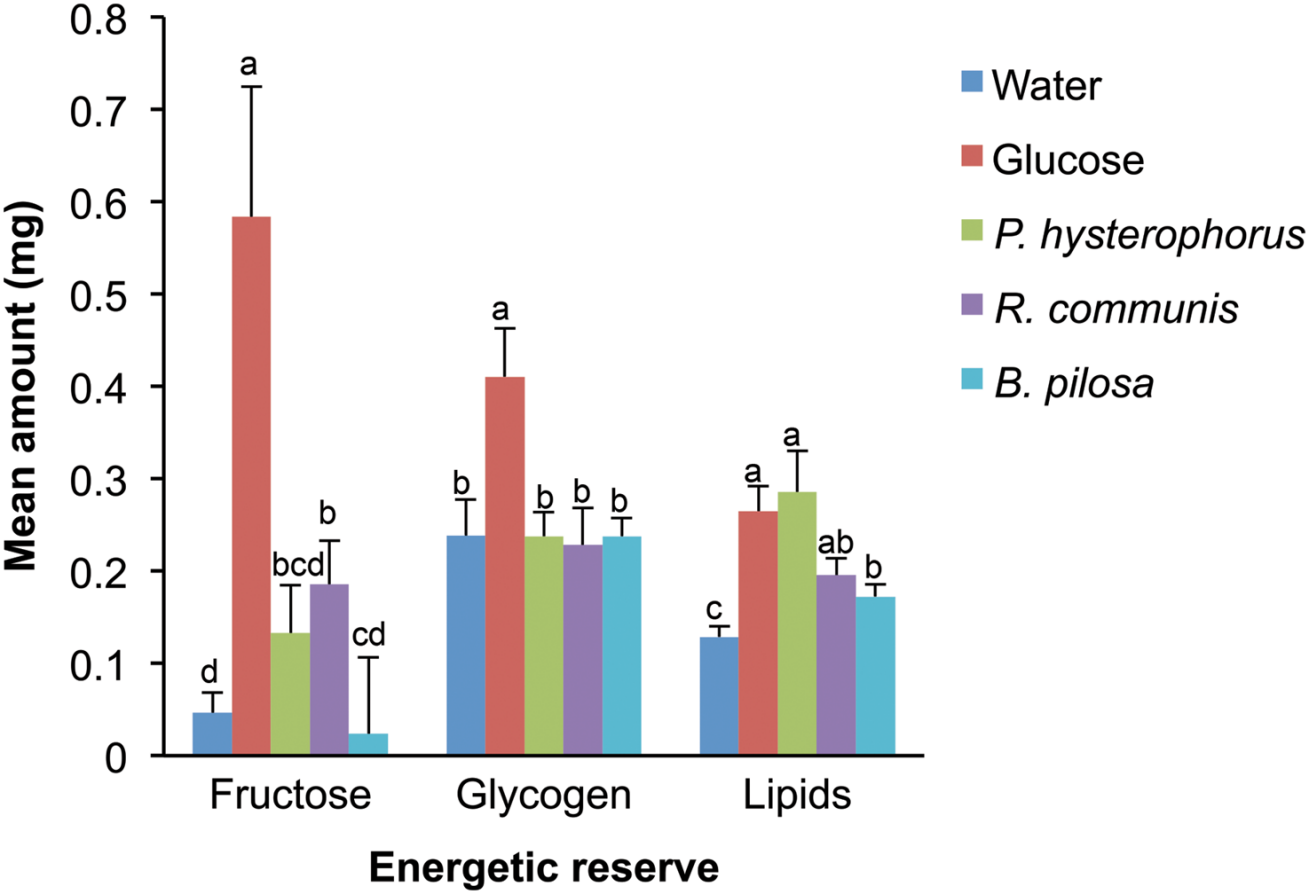
Energy reserves (sugar, glycogen and lipid content) of female *An*. *gambiae* exposed to different nutritional regimes after 7 days. Bars capped with different letters are significantly different.

Overall, there was a significant difference in the relative amounts of sugar as measured by the amount of total sugar and glycogen detected in the extracts of mosquitoes held on the five food regimes (Kruskal-Wallis chi-squared = 29.2495, df = 4, *P* < 0.001 and Kruskal-Wallis chi-squared = 11.9985, df = 4, *P* < 0.05 respectively). The amount of sugar detected was highest in mosquitoes held on 6% glucose solution followed by those held on *R*. *communis*, *P*. *hysterophorus*, *B*. *pilosa*, and water, in decreasing order (Fig 3). The amounts of sugar detected in mosquitoes held on *P*. *hysterophorus* and *B*. *pilosa* were not significantly different from those held on water. Apart from mosquitoes held on 6% glucose, the amounts of glycogen were not significantly different across the other four food regimes (Fig 3). On the other hand, lipid reserves were significantly different across the different food regimes (Kruskal-Wallis chi-squared = 19.0693, df = 4, *P* < 0.001), with mosquitoes held on *P*. *hysterophorus* and 6% glucose having the highest amount of lipids. Lipid content of mosquitoes held on *P*. *hysterophorus* differed significantly compared to those on *B*. *pilosa* but not on *R*. *communis* (Fig 3).

Of the four sugars (mannose, glucose, sucrose and maltose), identified from the mid-guts of mosquitoes (Table 3), mannose was not present in the mid-guts of mosquitoes fed on glucose solution but was detected in the mid-guts of those fed on the three plants. The amount of glucose detected was comparable in mosquitoes fed on 6% glucose solution, *R*. *communis* and *P*. *hysterophorus* while sucrose and maltose were relatively higher in mosquitoes fed on *R*. *communis* and *P*. *hysterophorus*. However, no significant difference was detected in the amounts of the three sugars between the three plants and glucose solution (Table 3). Only sucrose and maltose were detected in trace amounts in the mid-guts of mosquitoes held on water only, hence the results of this food regime were not included in the statistical analysis. Since we unexpectedly detected sucrose and maltose in the mid-gut of mosquitoes held on glucose solution, we further analysed the sugar content of the commercial glucose used to feed the mosquitoes. Our results showed that the commercial product had impurities of these sugars as follows: Glucose = 94.3%; Sucrose = 0.7%; and Maltose = 5.0%.

**Table 3:** Total amount of specific sugars present in *An*. *gambiae* mid-gut after feeding on different food regimes.

Type of sugar	Food regime	Mean amount ± SEM (ng)	CI	*P*-value
Mannose	*P*. *hysterophorus*	55.44 **±** 11.252	-29.81–140.65 (a)	0.237
	*R*. *communis*	32.41 **±** 3.379	-69.12–101.34 (a)	0.928
	*B*. *pilosa*	16.14 **±** 1.911	-52.85–117.61 (a)	0.634
Glucose	*P*. *hysterophorus*	73.80 **±** 10.536	-77.01–141.61 (a)	0.782
	*R*. *communis*	76.35 **±** 9.440	-110.48–108.14 (a)	0.743
	*B*. *pilosa*	40.33 **±** 5.726	-74.46–144.16 (a)	1.000
	Glucose solution	81.50 **±** 0.545	(a)	
Sucrose	*P*. *hysterophorus*	7.42 **±** 1.014	-35.87–34.98 (a)	1.000
	*R*. *communis*	24.61 **±** 4.842	-37.72–33.12 (a)	0.473
	*B*. *pilosa*	5.56 **±** 0.018	-18.68–52.18 (a)	0.997
	Glucose solution	7.86 **±** 0.084	(a)	
Maltose	*P*. *hysterophorus*	114.96 **±** 4.416	-19.97–221.55 (a)	0.105
	*R*. *communis*	130.19 **±** 15.186	-87.57–153.95 (a)	0.06
	*B*. *pilosa*	47.35 **±** 5.865	-4.74–236.78 (a)	0.815
	Glucose solution	14.17 **±** 0.057	(a)	

CI = confidence interval; CI marked with different letters are significantly different.

## Discussion

Our results show that mosquitoes survived moderately better on *R*. *communis* and *P*. *hysterophorus* as compared to *B*. *pilosa* and water, though the two did not perform as well as glucose solution. The results further revealed that sugar content was highest in mosquitoes held on glucose solution, followed by *R*. *communis* and *P*. *hysterophorus*, with lower levels recorded in those held on *B*. *pilosa* and water. With the exception of *An*. *gambiae* held on glucose solution, glycogen reserves did not differ significantly between the other four food regimes. On the other hand, mosquitoes held on *P*. *hysterophorus* had the highest lipid content, comparable to those held on glucose, with *B*. *pilosa* and water having the lowest lipid contents. Total energy reserves in terms of sugar, glycogen and lipid did not differ significantly between mosquitoes held on *P*. *hysterophorus* and *R*. *communis*. While these results corroborate our previous results, indicating that *R*. *communis* had the highest sugar content followed by *P*. *hysterophorus* while *B*. *pilosa* had the least sugar content [31], they also underpin the importance of plant sugars in *An*. *gambiae* survival. In insects, daily energy expenses are met through three essential ingredients: lipids, carbohydrates and to a lesser extent proteins [43]. Sugars provide a ready source of metabolic energy while glycogen and lipids are stored forms of energy in the fat body and are mobilized when insects are faced with food scarcity. The energetic threshold and critical egg load determine the need for feeding and the path of energy allocation in insects [44,45]. Our results point to energy-dependent survival as mosquitoes held on *B*. *pilosa*, which has the least amount of available sugars, resulted in the shortest life span, though significantly higher than those held on water. These findings may have significant ecological implications since in nature mosquitoes forage for sugars from plant nectars. Therefore, we suggest that should *P*. *hysterophorus* be able to completely replace *B*. *pilosa* in malaria endemic area [46], the life expectancy of the malaria vector may be extended, resulting in sustained and/or increased malaria transmission.

Interestingly, fructose, which is normally used as an indicator for recent sugar feeding [47], was not detected in the mid-guts of mosquitoes held on the three plants. This could have been possibly due to rapid absorption of this sugar by female *An*. *gambiae* as demonstrated by Gary [48]. However, mannose was detected only in mosquitoes that had fed on the three plants but not in those that had fed on glucose solution and water only, providing evidence of actual nectar feeding in mosquitoes held on these plants. When the sugar contents in mosquitoes feeding on the three plant species were compared, the amounts of mannose were highest in mosquitoes that had fed on *P*. *hysterophorus*, while glucose, sucrose and maltose were highest in *R*. *communis*. Interestingly, the quantities of glucose, sucrose and maltose found were comparable in mosquitoes held on *P*. *hysterophorus* and *R*. *communis*, which explains the near similarity of their survival on these diets. Overall, because *P*. *hysterophorus* is an aggressive invasive weed, commonly found in malaria-endemic areas, and is known to displace other plant species including edible ones in Africa [12,14,46], it may present malaria vectors with more resource opportunities for their sustained survival even where a blood source is not readily available.

Notably, the findings of this study on the survival of *An*. *gambiae* on *P*. *hysterophorus* and gut sugar contents differ with those reported by Manda et al. [15]. While in the latter study, mosquitoes exhibited poor survival on this plant, with a median of 4 days, with subsequent analysis showing poor sugar content compared to *R*. *communis* with relatively high survival rates (median 10 days) and high sugar content, the converse is demonstrated here. This discrepancy can be attributed to a number of factors including; 1) the use of intact plants in the current study as opposed to cuttings in the study by Manda et al. [15], 2) differences in environmental conditions and geographical locations of the plants used in the study by Manda et al. [15] and the current one, and 3) differences in the experimental conditions given that the current experiments were carried out under more controlled laboratory conditions.

The fact that *P*. *hysterophorus* produces the highly toxic compound parthenin [28] has raised considerable concern among governments and scientific agencies in East Africa and beyond, mainly due to its toxic effects on both livestock and humans [12]. Interestingly, in our study parthenin did not exert the same toxic effects on mosquitoes as the alkaloid ricinine when presented in the same concentration. On the other hand, survival of mosquitoes on phenylheptatriyne, the metabolite produced by *B*. *pilosa*, compared favourably with glucose. Similar findings were reported by Wachira et al. [49], who demonstrated that ricinine was highly toxic to *An*. *gambiae* while parthenin and phenylheptatriene did not show any toxicity. While these results support the hypothesis of fitness-related benefits as the basis for host plant selection [15], they also serve as possible indicators of a resilient vector species capable of tolerating certain cytotoxic substances in the environment, including highly toxic poisons produced by an invasive plant species.

Our results further indicate that *An*. *gambiae* females can tolerate and/or possibly clear from their system much of the toxic substances ingested from the three plant species. Given that survival on parthenin and phenylheptatryine solutions was as good as that on glucose alone, these results point to a potentially resilient malaria vector capable of detoxifying itself of substances toxic to vertebrates, particularly parthenin which is toxic even to human beings. This hypothesis of self clearance of the toxic substances is further supported by our results which show that the plant secondary metabolites could only be detected up to 24 hrs, coinciding with the time when the metabolite solutions were removed and replaced with glucose solution. No traces of these compounds were detected after 48 and 72 hrs. Another possible explanation of the observed *An*. *gambiae* tolerance could be due to presence of gut microflora that are capable of metabolizing plant secondary metabolites, as has been demonstrated in other herbivores, such as desert wood rats (*Neotoma lepida*) [50]. On the other hand, mosquitoes suffered high mortality when exposed to ricinine, a highly toxic and carcinogenic compound [29], and the compound is clearly toxic to the mosquitoes at the dose used in our experiment. These results indicate that the levels of ricinine acquired by mosquitoes feeding naturally on *R*. *communis* might not be as lethal. Our finding that acetone did not have significant effect on the survival of *An*. *gambiae* when mixed with glucose solution shows that the mortality rates recorded for different metabolites is due to their toxic effects.

Our results also show that mosquitoes held on *P*. *hysterophorus* were able to build greater lipid reserves as compared to the other plant species provided. Lipids are the more efficient form of stored energy due to their high caloric value [51] and can be synthesized from sugar meals by mosquitoes [52]. This explains the high lipid content observed in mosquitoes held on glucose solution. However, given that sugar reserves in *P*. *hysterophorus* are lower than in *R*. *communis* [31], this raises the possibility that mosquitoes also obtain lipids directly from feeding on this plant. Alternatively, it is possible that *P*. *hysterophorus* contains some unique sugars that were not identified in this study or higher amounts of certain sugars such as mannose, which can be metabolised to lipid reserves. Lipids play a critical role in insects including: serving as a source of energy, functioning as cell membrane components, and acting as signalling-pathway modulators and emulsifying agents. They are the major source of energy for developing embryos in the eggs [53] and have been implicated as playing a role in the outcome of mosquito-*Plasmodium* interactions [54]. It would be interesting to further evaluate the potential of *P*. *hysterophorus* to provide a ready lipid source for Afrotropical malaria vectors.

Overall, the survival of *An*. *gambiae* on the three plant species was lower than their survival on glucose solution alone and glucose solution laced with the respective plant metabolites despite the fact that mosquitoes may be exposed to additional sugars in the plants. The availability and concentrations of nectar sugars play a vital role in determining the success of nectar feeding in herbivorous insects [55,56]. As such, the low survival rates observed in mosquitoes held on the plants compared to those held on glucose solution can be attributed to either low sugar concentration as opposed to the type of sugars ingested, as evidenced by the different survival rate which correlates to the amounts of sugar present in the three plant species. In addition, the mosquito’s proboscis might not be well adapted to pierce the cellular walls of succulent plants such as *P*. *hysterophorus* and *B*. *pilosa*. On the other hand, *R*. *communis* has extrafloral nectarines provided in the form of plant exudates, thereby possessing more easily available sugars to the mosquitoes than the other two plant species.

## Conclusion

Our results highlight the potential epidemiological implications of the highly invasive and aggressive weed *P*. *hysterophorus*, which enhances the energy reserves of *An*. *gambiae*, suggesting the need for consolidated efforts to curb its spread, especially in malaria endemic areas. Given that invasive species like *P*. *hysterophorus* can suppress or even replace less competitive indigenous species that might be less favourable host-plants for disease vectors, the spread of introduced plants could subsequently lead to higher disease transmission. Hence in certain instances risk-analysis modelling of invasive plants also needs to include the human-health dimension.
